# I am a child, i learn through play; play-based character education

**DOI:** 10.3389/fpsyg.2025.1613650

**Published:** 2025-09-24

**Authors:** Levent Görün, Hande Baba Kaya, Rumeysa Alper, Mustafa Koç, Aylin Çelen, Selahattin Akpınar, Şirin Pepe, Yeşer Eroǧlu Eskicioǧlu

**Affiliations:** ^1^Department of Physical Education and Sports Teaching, Faculty of Sport Sciences, Duzce University, Duzce, Türkiye; ^2^Department of Educational Sciences, Faculty of Education, Duzce University, Duzce, Türkiye; ^3^Department of Physical Education and Sports Teaching, Faculty of Sport Sciences, Abant Izzet Baysal University, Bolu, Türkiye; ^4^Department of Sports Management, Faculty of Sport Sciences, Duzce University, Duzce, Türkiye

**Keywords:** character education, behavior change, child, play, character

## Abstract

Character is the set of traits that integrate moral knowledge, emotion, and action, enabling individuals to act as moral agents. The purpose of this study is to examine the effect of a play-based character education program on children's behaviors. This study was designed according to the exploratory sequential design, one of the mixed method designs. The study group consisted of 40 children who received high scores from the behavior assessment scale for children applied to first-grade students studying in a primary school in the central district of Düzce. Within the scope of the study, the Behavior Assessment Scale for Children developed by Chuang was used to make a general evaluation of children's behaviors and to collect information. The Turkish validity and reliability of the scale was conducted by Sişman. The data obtained within the scope of the research were coded into the SPSS 26.0 package program and *t*-test analysis for unrelated samples was performed to determine the effect of the application within the scope of the game-based character education program on the dependent variable. As a result of the analysis, it was seen that the game-based character education program applied to the experimental group had a significant effect on different groups (experimental-control), and the eta square statistic value of this effect had a large effect size.

## 1 Introduction

Character is defined as personal traits or virtues that ensure the consistency of moral actions. This concept encompasses cognitive, affective and behavioral aspects, including moral knowledge, moral feeling and moral action. Berkowitz (2002) defined character as the sum of psychological traits that influence an individual's ability to function morally. Good character can be defined as the ability to know the good, will the good, and act the good (Yildirim and Gürpinar, 2023). In a similar source, character is defined as complex set of psychological traits that enable an individual to act as a moral actor (Berkowitz and Bier, 2004). Behavior regulation constitutes the majority of character education. Behavior regulation is one of the types of self-regulation. In order for the learning process to take place successfully in children who have just started school, it is very important to have behavior regulation skills in terms of both academic and social performance. In schools, children need to be able to control their behavior in order to direct their attention to certain tasks. A problem in a child's behavior regulation skills will affect his/her academic success and success in social relationships (Ponitz et al., 2008).

The beginning of school is a critical period for the formation of self-regulation skills. A 6 year-old child with well-developed self-regulation skills exhibits behaviors such as waiting for his/her turn, sharing course materials, controlling impulsive behaviors, communicating appropriately, following the routines of school life, and following classroom rules (Blair, 2002; Meyers and Berk, 2014). Children with high self-regulation skills can control their thoughts, emotions and behaviors. Children's ability to exhibit these desired behaviors includes mental processes such as sustaining attention, impulse control, the ability to flex their thoughts and behaviors, and the ability to exhibit purposeful desired behaviors (Meyers and Berk, 2014; Welsh et al., 2008).

There are different factors that are effective in the acquisition of self-regulation skills on behaviors. Play is one of the most important factors that provide self-regulation skills (Bodrova et al., 2013; Savina, 2014; Whitebread et al., 2009). In play, the child exhibits behaviors such as voluntarily participating in the rules, determining his/her own limitations without being under pressure, designing the rules of the game with his/her friends, agreeing on the roles in the game and fulfilling his/her duties. Thus, they gain self-regulation skills (Savina, 2014). Elias and Berk (2002) stated that children tend to behave in accordance with social norms in play. This process is referred to as the time when self-regulation begins (Bodrova et al., 2013).

According to Erikson, when children play, they create their own social space where they follow the rules of behavior of the group they cooperate with. In this social space, which is intertwined with real life, children learn both to behave in accordance with social rules and to transfer these accepted behaviors to their real lives (Meyers and Berk, 2014). In play, children set limits on their own behaviors, which is very important in terms of distinguishing between desired behaviors and undesired behaviors through their own experiences. Because in real life, children unconsciously obey the rules that adults tell them without understanding the reasons. Prohibitions and rules imposed from outside are abstract concepts that are difficult for children to understand. Children can only concretize the rules they encounter in play and learn from them. Furthermore, research shows that play behavior varies by developmental stage. While younger children often engage in parallel or imaginative play, children in the early elementary years (ages 6–7) increasingly prefer structured, rule-governed games that promote cooperation and social negotiation (Wood, 2013; London Fleer, 2017; Dogan, 2021). These types of games are particularly effective in character education, as they allow children to experience fairness, empathy, and emotional control in contextually meaningful ways (Whitebread et al., 2017). Thus, the play-based model implemented in this study was designed specifically in alignment with the developmental needs of this age group, maximizing its relevance and potential impact on behavior.

Good character does not happen automatically; it is developed over time through teaching, example, learning and practice. Especially for today's children, character education is of vital importance (Pala, 2011). Character education can be defined as a conscious and pre-planned effort by schools and governments to instill in students basic moral values such as care, honesty, justice, responsibility and respect (Singh, 2019). This education involves the entire school community, including the school culture and curriculum, and is important for the acquisition of important qualities such as justice, care and compassion. It also contributes to the success of democratic societies by promoting values such as civic virtue, respect for the law, and a sense of common good (Singh, 2019). The most general meaning of character education is growing as a human being (McGrath, 2018). Although there are different approaches to how character education programs should be designed, the most important accepted idea is that this education should be provided within the school and curriculum (Berkowitz and Bier, 2004; Lapsley and Narvaez, 2006; Pala, 2011; Singh, 2019). Character education needs to be high-quality and comprehensive, but it is often difficult to find schools that meet all of these standards (Berkowitz, 2002). For this reason, it is very valuable to carry out activities that support character education outside the school curriculum.

When the literature is examined, it is understood that there are many programs based on character education, but there is no character education that emphasizes play (Ekşi, 2003; Gökçek, 2007; Turan, 2014; Yilmaz, 2021). Play plays a critical role in personality development. Because it encourages human relations, supports creativity, increases the joy of living and supports learning. For children, gaining direct experiences enables them to build knowledge, develop abstract thinking and generalize to new situations. Play is also an effective tool for teachers to introduce new ideas and concepts to children (Burriss and Tsao, 2002). With these aspects, it is understood that play is one of the most important elements that support character development (Deger, 2024). Especially competition-style games teach values such as playing according to the rules, appreciating the winner, reacting to defeat in moderation, and experiencing joy in a controlled manner (Yaliz, 2011). Therefore, integrating character education with play-based methods not only meets children's developmental needs but also increases the effectiveness and sustainability of value acquisition (Lickona, 1996; Berkowitz and Bier, 2005). For this reason, it can be predicted that providing character education on the basis of games will lead to positive results in gaining desired behaviors. There is a need for a program in this context.

This study focuses specifically on first-grade primary school children (age 6–7) and aims to examine the impact of a structured play-based character education program on observable behavioral outcomes over an 12-week implementation period.

## 2 Method

### 2.1 Research model

**Figure 1:**
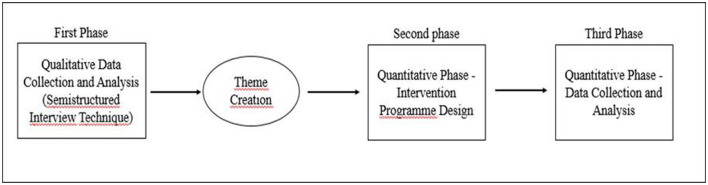
Schematic view of the research design.

This study was planned according to the exploratory sequential design, one of the mixed method designs. In the first stage of the mixed method, qualitative data were collected and the study group and game-based character education program were formed. In the second stage of the research, a randomized model with pretest-posttest control group was used. In this model, there are two groups formed by random assignment. One of the groups is the experimental group and the other is the control group. Both pretest and posttest data were collected from the groups and the effect of the independent variable was examined (Karasar, 2016; Creswell, 2021; Creswell and Creswell, 2023; Karasar, 2023) (Figure 1).

### 2.2 Participants

The research was conducted in the most crowded school with a socio-economically diverse student population in Düzce Province. In order to determine the study group, opinions were collected from first grade teachers about the undesirable behaviors they observed in students. The collected qualitative data were coded by the researchers and presented in Table 1. Within the framework of the themes and sub-themes, the scope framework of the game-based character education to be implemented was determined. In the quantitative part of the study, criterion sampling method, one of the purposive sampling methods, was used to determine the experimental and control groups. The criterion for inclusion in this study was getting high scores from the behavior assessment scale. First grade teachers were asked to fill out the behavior assessment scale for a total of 180 children. Accordingly, 40 children with the highest scores on the scale were determined as the study group. The study group was divided equally into two groups (experimental-control) using the random assignment method in accordance with the pretest-posttest control group model. In the study, which initially started as 20 experimental and 20 control groups, children who did not participate in the implementation process were excluded from the study. When the implementation process was over, the final measurements were taken and the study was finalized with 15 child participants in the experimental group and 19 child participants in the control group.

**Table 1:** Teachers' views on the undesirable behaviors they observe in students.

**Theme**	**Sub theme**	**Coding**
Undesirable behaviors	Congratulations and appreciation	Congratulating a friend on winning Showing measured joy when you win Reacting in moderation when losing Appreciating a friend for good behavior
	Dress and appearance	Wearing clean clothes Dress appropriately for the activity Clean and smelling good
	Compliance with rules	Keeping hair neat Waiting in line Following game instructions Not violating the rules of the game No unfairness or cheating
	Communication	Communicating positively with other people Not swearing or insulting (not saying bad words) Being respectful toward other people Not to harm anyone Speaking by taking the floor Listening to a friend talking Listening while the teacher speaks
	Awareness of duties and responsibilities	To fulfill the assigned task Follow the instructions/steps necessary to complete the assigned tasks Being responsible for oneself and not blaming others
	Environmental cleanliness awareness	Proper placement of event materials Leaving the locker room clean Not throwing garbage on the ground Putting the received material in its place

### 2.3 Data collection

#### 2.3.1 Quantitative data collection

The Behavior Assessment Scale for Children (BASC) used in the study is based on self-regulation theory and aims to assess the child's behavior (Chuang et al., 2016). Teachers are aware of children's development and behavior due to their profession (Sişman et al., 2021). Therefore, it was deemed appropriate for teachers to fill out the Behavior Assessment Scale for Children. Before the scales were filled in, an informative meeting was held with the participation of teachers and researchers about the purpose and scope of the scale.

The Behavior Assessment Scale for Children was developed by Chuang et al. (2016) to make a general assessment of children's behaviors. The Turkish validity and reliability of the scale was conducted by Sişman et al. The scale consists of 17 items and a three-factor structure (cognitive, emotional, and behavioral factors) that is easy to understand and apply. The scale is a 3-point Likert scale and is scored between 0 and 2; 0 points = no match, 1 point = moderate match and 2 points = relatively strong match. A higher score indicates lower performance. The CVI score of the original scale was 0.98. The result of confirmatory factor analysis shows that GFI = 0.90, RMSR = 0.03, RMSA= 0.06 and CFI = 0.94. Cronbach's alpha coefficients of the subscales were reported to range between 0.78 and 0.82 (Chuang et al., 2016). For the Turkish version of the scale, Cronbach's alpha coefficient was found to be 0.73 in the forms filled out by teachers (Sişman et al., 2021). Within the scope of this study, the Cronbach's alpha coefficient of the scale was found to be 0.74 and the internal consistency level was found to be good.

#### 2.3.2 Qualitative data collection

In the qualitative data collection phase of the study, semi-structured interview technique was used to record individual interviews with teachers in the institutions where they work. The ethics committee approval required for the study was given by Düzce University scientific research and publication ethics committee (Number: E-78187535-050.04-472663).

Semi-structured interview question;

1. What are the undesirable behaviors you encounter in your students?

### 2.4 Play-based character education program content and implementation process

The play-based character education program prepared by the researchers was implemented for a total of 12 weeks. The interventions were conducted for a total of 36 sessions of at least 60 min and 3 days a week. While preparing the content of the game-based character education program, a lesson model based on young age groups was created. The lesson started with order exercises, which are the basis of physical education, and continued with games and ended with order exercises again and a motivational speech by the researcher. The reason for game-based character education is to concretize the concept of character education, which is abstract for children in the 6–7 age group, who are first grade students, through games. This study chose a structured and goal-oriented play-based learning approach. This approach was guided by the developmental needs of 6 and 7-year-olds who benefit from activities with clear rules and objectives.

This approach aims to help ensure the permanence of behavior change. Considering that this age group learns best through in-game activities, character education content was combined with game content and children were given hands-on activities. The reason for starting the lessons with organization exercises is that these exercises help children learn how to take commands, follow instructions and act in harmony as a group. Such exercises make it easier for children to follow the teacher's instructions and to understand how to behave harmoniously with their peers in the group.

**Figure 2:**
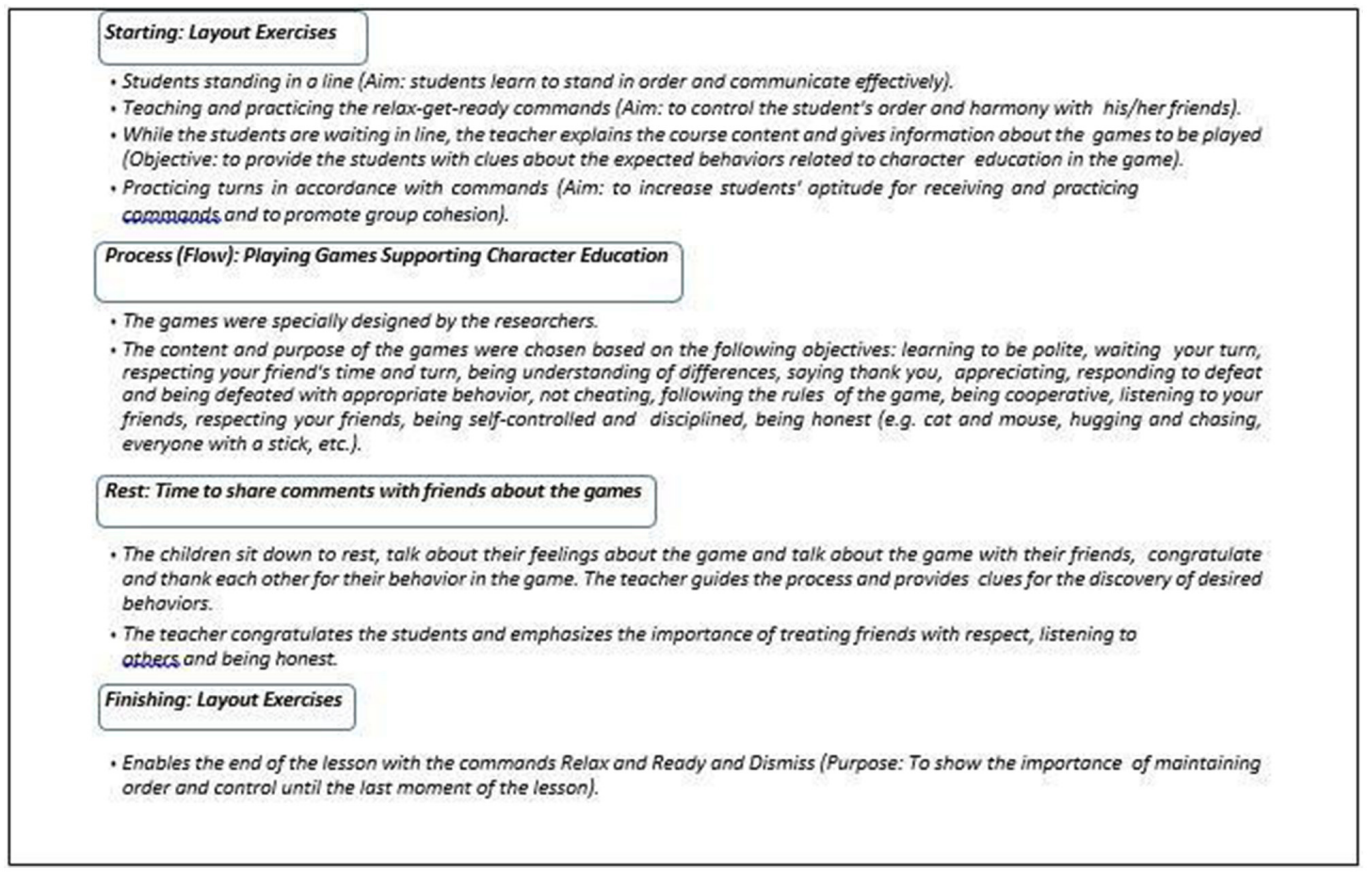
Flow chart and content of the play-based character education program.

While creating game-based character education programs, six main themes were formed by processing the qualitative data obtained from the preliminary interviews with teachers and the main themes were explained with items within themselves. A game-based character education program was developed for the explained items and training was carried out within the framework of the developed program. The six main themes are: congratulations and appreciation, dress, and appearance, compliance with in-game rules (awareness of following the rules), communication, awareness of duty and responsibility, and awareness of environmental cleanliness. The flow chart of the game-based character education program created in the light of these data is shown in Figure 2.

In the selection of games, first of all, games that were suitable for the age group of the children and that they could enjoy were prioritized. Among these, preference was given to games that included content supporting the development of core character education components such as honesty, respect, responsibility, fairness, helpfulness, and cooperation, and that were believed—based on teacher opinions—to contribute to the development of positive behaviors. During the implementation phase, the role of the implementing teachers was not limited to introducing and supervising the games; rather, they actively engaged in motivating students, providing positive reinforcement, and guiding behavioral reflection. Teachers used encouraging language such as “Great teamwork!”, “That was a very fair decision”, or “I appreciate how you helped your friend” to reinforce desirable behaviors in the moment. When students demonstrated actions aligned with character values, teachers made those behaviors visible and meaningful through immediate verbal feedback and praise. Moreover, in situations where negative behaviors emerged, teachers facilitated reflective conversations by asking guiding questions like “What could we do differently next time?” or “How did that make your friend feel?” to promote empathy and self-awareness. In this way, the teacher-student interaction became a crucial component of the program, transforming each game into an opportunity for social-emotional learning and internalization of values.

### 2.5 Analytical strategy

The suitability of the use of parametric tests in analyzing the data obtained within the scope of the research was examined. Independent Samples *t*-test, one of the parametric tests, is used to test whether there is a significant difference between the averages of two independent groups. The assumptions for this test are that the total mean scores are at least in the interval scale suitable for comparison, the variable data show normal distribution characteristics in both groups, the two groups are independent from each other, and the variances of the groups are expected to be equal (Can, 2019; Büyüköztürk, 2014; Field, 2009). Among these assumptions; when the assumption that the distribution of the variable data in both groups shows normal distribution characteristics is met, the condition of having at least 10 samples (n1 ≥ 10 and n2 ≥ 10) in each group, the number of samples required for parametric tests can be stretched (Sümbüloglu and Sümbüloglu, 2007; Cevahir, 2020). In this direction, the skewness and kurtosis values of the pre-test and post-test scores of the control and experimental groups were examined to examine the appropriateness of the assumptions and are shown in Table 2.

**Table 2:** Shapiro-wilk, skewness and kurtosis test results of pre-test and post-test scores of control and experimental groups.

**Groups**	**Measurement**	***n***	**W**	**Skewness**	**Kurtosis**	***p***
Control groups	Pre-test	19	0.956	−0.010	−0.695	0.495
	Post-test	19	0.962	0.014	−0.481	0.619
Control groups	Pre-test	15	0.926	−0.783	0.513	0.235
	Post-test	15	0.949	−0.616	0.473	0.509

It is seen that the skewness and kurtosis values of the scores obtained from Table 2 are between +1.0 and −1.0 values accepted for the field of social sciences (Mangiafico, 2016; Büyüköztürk et al., 2016). In addition, whether the values show a normal distribution was also examined with the Shapiro-Wilk test, one of the normality tests. Since the number of participants in the study was less than 50, it was thought that the use of the Shapiro-Wilk test was more appropriate (Büyüköztürk, 2014). When the results of the Shapiro-Wilk test were examined, it was accepted that the distribution obtained from the pre-test and post-test measurement scores in the control and experimental groups was normal. When the *p* value is greater than 0.05 in normality tests, it can be said that the scores do not deviate excessively from the normal distribution at this significance level and are suitable for parametric tests (Büyüköztürk, 2014). After it is accepted that the normality assumption of the measurement data of the dependent variable is met for the groups, another assumption is that the variances of the dependent variable must be homogeneous for each sample (Büyüköztürk, 2014). In this context, Levene's test was used to examine the variance homogeneity of the groups. The equality of variances of the pre-test and post-test scores of the control and experimental groups were examined with “Levene's Test for Equality of Variances” and shown in Table 3.

**Table 3:** Levene's test results for the pre-test and post-test scores of the control and experimental groups.

	**Measurement**	***n***	**Sd1**	**Sd2**	***F***	***p***
Scale	Pre-test	34	1	32	067	0.797
	Post-test	34	1	32	012	0.915

In Table 3, no statistically significant difference was found between the variances of both the pretest and posttest scores of the control and experimental groups. When the values obtained from the pre-test (*F* = 0.067, *p* < 0.5) and post-tests (*F* = 0.012, *p* < 0.5) of the scale are analyzed, it can be said that the variances of the pre-test and post-test measurements of the control and experimental groups are homogeneous. Therefore, it was found appropriate to use parametric tests in the study.

In the study, the dependent groups *t*-Test, which is one of the parametric tests, was used to determine the development of the control and experimental groups at the end of the application, and the independent groups *t*-Test was used to determine the difference between the post-tests of control and experimental groups after the application.

## 3 Findings

In the pre-tests of the control and experimental groups, *t*_(32)_ = 1.452, *p* > 0.05for the attention sub-dimension, *t*_(32)_ = 0.060, *p* > 0.05 for the emotion sub-dimension and *t*_(32)_ = 154, *p* > 0.05 for the self-control sub-dimension, there was no significant difference (Table 4). The fact that the pre-test scores of the control and experimental groups before the experimental procedure did not differ from each other indicates that the study groups were homogeneous. It is a desired result in order to test the effectiveness of the applied procedure. There was no significant difference between the total scores of the control and experimental groups, *t*_(32)_ = 0.932, *p* > 0.05. The fact that the pretest scores of the control and experimental groups did not differ from each other before the experimental procedure indicates that the study groups were homogeneous. It is a desired result in order to test the effectiveness of the applied procedure.

**Table 4:** Comparison of the pre-test scores of the control and experimental groups.

**Sub- dimensions**	**Groups**	**Measure**	***N***	***X***	***S***	**sd**	***t***	***p***
Attention	Experimental groups	Pre-tests	15	10.20	1.93	32	1.452	0.156
	Control groups	Pre-tests	19	8.68	3.65			
Emotion	Experimental groups	Pre-tests	15	5.20	2.07	32	0.060	0.952
	Control groups	Pre-tests	19	5.15	1.98			
Self-control	Experimental Groups	Pre-tests	15	10.66	3.18	32	0.154	0.879
	Control groups	Pre-tests	19	10.52	2.14			
Total	Experimental groups	Pre-tests	15	26.07	5.37	32	0.932	0.359
	Control groups	Pre-tests	19	24.37	5.19			

**Figure 3:**
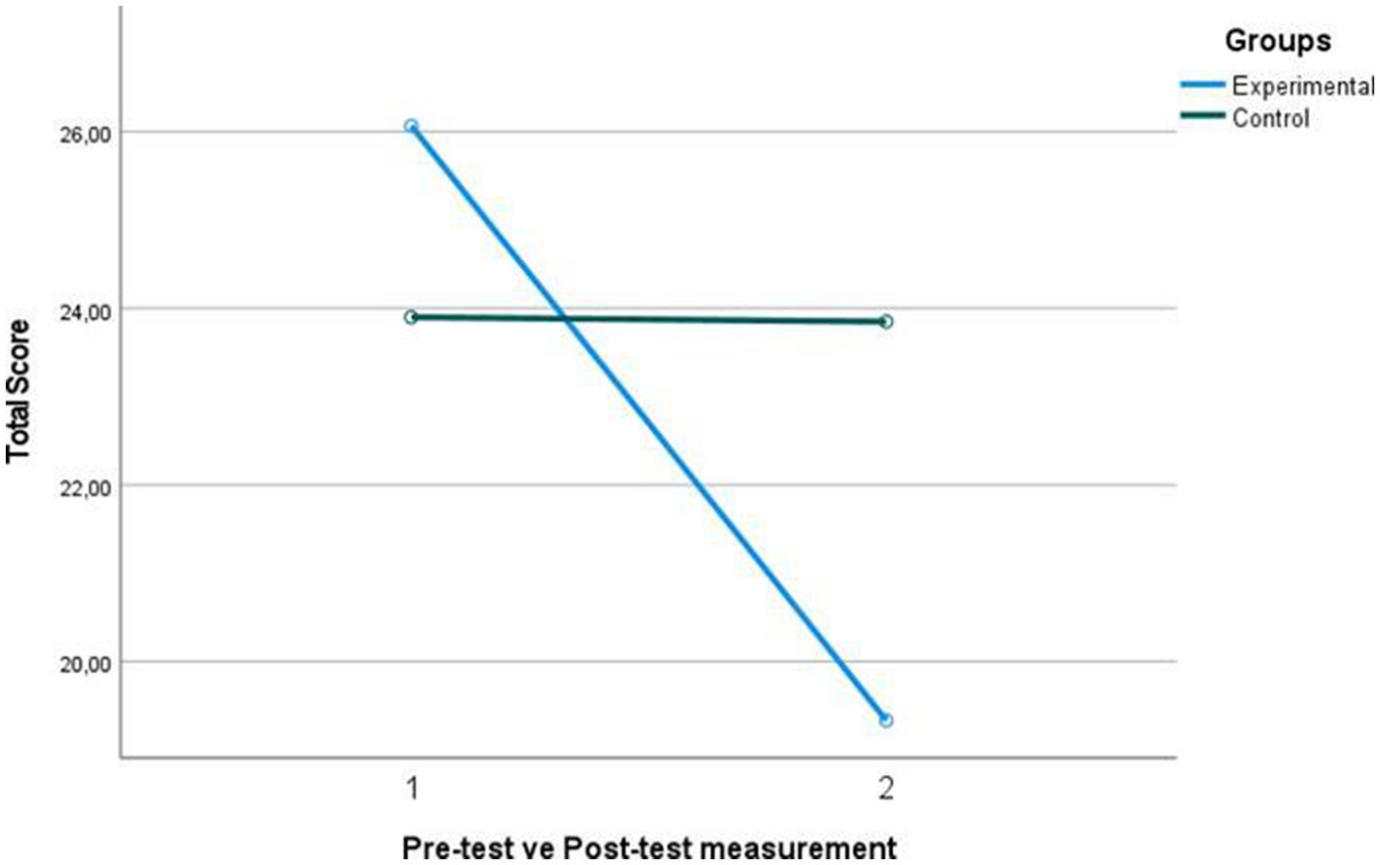
Graph of Pre-test and Post-test total BASC scores of control and experimental groups.

At the end of the play-based character education program of the experimental group, it is seen that there is a significant difference between the pre-test and post-test scores they received from the sub-dimensions of the BASC (Figure 3). While the average scores of the students from the attention sub-dimension of the scale before the experimental procedure was X = 10.20, the average of the scores they received from the attention sub-dimension after the experimental procedure was X = 7.20. While the average scores they received from the emotion sub-dimension were X = 5.20, the average of the scores they received from the emotion sub-dimension after the experimental procedure was X = 3.86. It is seen that while the average score they received from the self-control sub-dimension was X = 10.66, the average score they received from the self-control sub-dimension after the experimental process was X = 8.26. At the end of the game-based character education program, it is seen that the scores of the students in the experimental group decreased in all sub-dimensions of the BASC. In the control group, there was no significant difference between the pre-test and post-test scores they received from the sub-dimensions of the BASC. With this finding, it can be concluded that the game-based character education positively affected the behaviors of the students in the experimental group. At the end of the game-based character education program of the experimental group, it is seen that there is a decrease in the Total Scores Received from the BASC. While the average scores of the students from the scale before the experimental process was X = 26.06, the average of the scores they received from the scale after the experimental process decreased to X = 19.33. This finding shows that the game-based character education applied to the students showed more positive behaviors in the behavior assessment scale and the mean scores decreased (Table 5).

**Table 5:** The pre-test post-test means of the control and experimental groups' scale scores.

**Sub- dimensions**	**Groups**	**Measure**	***N***	**X**	**S**	**sd**	***t***	***p***
Attention	Experimental groups	Pre-tests	15	10.20	1.93	14	3.873	0.002^*^
		Post-test	15	7.20	3.16			
	Control groups	Pre-tests	19	8.68	3.65	18	−1.455	0.163
		Post-test	19	9.00	3.38			
Emotion	Experimental groups	Pre-tests	15	5.20	2.08	14	2.597	0.021^*^
		Post-test	15	3.86	1.41			
	Control groups	Pre-tests	19	5.15	1.97	18	−1.143	0.268
		Post-test	19	5.47	1.80			
Self-control	Experimental groups	Pre-tests	15	10.66	3.18	14	3.180	0.007^*^
		Post-test	15	8.26	3.30			
	Control groups	Pre-tests	19	10.52	2.14	18	1.824	0.085
		Post-test	19	9.84	2.50			
Total	Experimental groups	Pre-tests	15	26.06	5.38	14	4.668	0.001
		Post-test	15	19.33	5.64			
	Control groups	Pre-tests	19	24.36	5.19	18	0.325	0.749
		Post-test	19	24.31	5.13			

^*^p < 0.05.

In the final measurements of the control and experimental groups, *t*_(32)_ = 4.556, *p* < 0.05 for the attention sub-dimension, *t*_(32)_ = 2.993, *p* < 0.05. for the emotion sub-dimension, and *t*_(32)_ = 2.170, *p* < 0.05. for the self-control sub-dimension. At the end of the game-based character education program, it is seen that the post-test scores of the experimental group from all sub-dimensions of the BASC decreased significantly compared to the post-test scores of the control group, in which no action was taken. There was a significant difference in the post-test scores of the control and experimental groups, *t*_(32)_ = −2.691, *p* < 0.05 In the post-test results obtained after the game-based character education program, the total scores of the experimental group (X = 19.33) were more positive than the total scores of the control group (X = 24.31) (Table 6). This finding can be interpreted that play-based character education has positive effects on children's behavior.

**Table 6:** *T-test* results of posttest score means of control and experimental groups.

**Sub- dimensions**	**Groups**	**Measure**	***N***	**X**	**S**	**sd**	***t***	***p***
Attention	Experimental groups	Post-test	15	7.20	3.16	32	4.556	0.001
	Control groups	Post-test	19	9.00	3.38			
Emotion	Experimental groups	Post-test	15	3.86	1.40	32	2.993	0.005
	Control groups	Post-test	19	5.47	1.80			
Self-control	Experimental groups	Post-test	15	8.26	3.30	32	2.170	0.038
	Control groups	Post-test	19	9.84	2.50			
Total	Experimental groups	Post-test	15	19.33	5.63	32	2.691	0.011
	Control groups	Post-test	19	24.31	5.13			

This finding indicates that the game-based character education program applied to the experimental group had a significant effect on different groups (experimental-control) [*t*_(32)_ = −2.691, *p* < 0.05, η*2* = 0.185], and the eta square statistic value of this effect has a large effect size. According to this, it can be said that 18.5% of the variance between the control and experimental groups was caused by the experimental process of game-based character education. On the other hand, the calculated Cohen's d value is 0.93, which indicates that the difference between the control group and the experimental group in the post-test mean scores of the BASC is 0.93 standard deviation.

## 4 Discussion

In this study, which aimed to reveal the effect of play-based character education on children's behaviors, it was observed that there were decreases in the undesirable behaviors of the children in the experimental group. Hasmarita et al. (2023) found that educational games positively affected the character development of primary school students. Melati and Suparno (2020) investigated the effects of game-based learning and found that 75% of the children were at a weak level of responsibility and there were no children at a high level of responsibility. After the game-based interventions, she found that children's character development increased by approximately 53% and they were included in the high-level responsibility category. Putra and Hasanah (2018) aimed to support character development in children with a traditional game played by children in Indonesia. As a result of the study, it was revealed that there was improvement in character components such as cultural literacy, religious values, cooperation, responsibility, honesty, self-confidence and curiosity. Similarly, Usta and Yorulmaz (2023) reported in their study that play-based education provided development in character components such as responsibility, patience and honesty in students. Izgar (2020) concluded that play-based education has a positive effect on the values of justice, friendship, honesty, patience, responsibility, solidarity, equality, and solidarity in primary school children. In addition, in the study, in which teachers‘ opinions were also taken, it was determined that play-based education provides students with concrete experiences in gaining values, students are eager to participate in play-based education and play with fun. Mashuri (2022) concluded that games have a great impact on strengthening the character of primary school students. In their study, Kiliç and Babayigit (2017) determined that there was a decrease in the awareness of love, respect and responsibility in primary school students, and that students had socialization problems and were egocentric. In today's society, communication problems between people, lack of tolerance, and distancing human values such as love, respect and cooperation continue to increase. In order to prevent all these negativities, human values should be taught to children at a very young age and these values should be adopted permanently (Senek, 2018). While teaching character and values to children, it is necessary to create a suitable environment where they can learn by doing and experiencing and to provide them with the opportunity to play games. Children acquire many human values such as respect, honesty, helpfulness, happiness and tolerance while playing games. Children who play games at home and at school have fun while playing and learn while having fun. Therefore, a more effective, easy and permanent values education can be provided through games (Erdal, 2019). In addition, it is thought that abstract values such as responsibility and respect, which are difficult to teach to students, can also be taught through games (Ilgar and Tutumlu, 2021). Within the scope of this study, it was seen that the play-based character education program had positive effects on attention, emotion and self-control skills, which are the sub-dimensions of the behavior assessment scale. When the literature is examined, the positive effects of the game-based character education program on attention development have been revealed in various studies. White and Warfa (2011) stated that character education increases attention and focus in the classroom environment by improving social interaction among students. The interaction between character education and cognitive development suggests that games can stimulate cognitive processes by reinforcing social values (Hasan and Husein, 2024). This dual benefit emphasizes the importance of integrating character education into game-based learning frameworks. Nurindarwati (2022) noted that character education is important for developing children's cognitive capacities and moral frameworks. Play-based character education plays an important role in shaping children's cognitive and emotional development (Bul et al., 2015; Pohl and Mottelson, 2022). In their study, Bachen et al. revealed that play-based education contributes to students' emotional development (Bachen et al., 2012, 2016). Another study showed that play-based learning models support cooperation and teamwork, self-confidence and improve emotional skills among primary school students (Susanto, 2022). Games support children's learning through emotional responses such as curiosity and achievement and increase their motivation (Sagimbayeva, 2022). It shows that play-based interventions offer children opportunities to develop self-control in a fun and structured environment. For example, Pesce (2020) found that physical activities increase self-control and emotional regulation. Williams and Berthelsen (2019) reported that musical games improve self-regulation, especially in disadvantaged children. In addition, Zachariou (2023) discovered that games improve self-control skills such as attention and mental flexibility. Play is also an effective tool for teaching emotional regulation and coping strategies (Dehghan, 2016). Play-based character education programs are an effective method to encourage attention development in children and to support their self-control, emotional and social development. When the results of the research and the related literature are evaluated together, it can be concluded that play-based programs have positive effects on the character development of primary school children.

### 4.1 Conclusion and recommendations

In this study, the character development of first grade primary school students was tried to ensure through play-based activities. It was concluded that the experimental group students who were given game-based character education improved their behaviors in attention, emotion and self-control sub-dimensions of the “Behavior Rating Scale for Children” throughout the process. No statistically significant change was observed in both the sub-dimensions of the scale and the total scores of the scale in the control group students who were not subjected to the experimental process. In addition, it was determined that the experimental group students showed more positive behavioral changes between the experimental group that received game-based character education practices and the control group that did not receive any treatment. As a result, it was determined that play-based character education of primary school students showed development on the basis of attention, emotion and self-control.

The following suggestions can be made for future studies.

- This study was limited to 12 weeks. In future studies, the game-based character education process can be conducted over a longer period of time.
- The study was limited to first grade primary school students. The effects of similar programs supporting character education on different age groups can be examined.
- Activities that support character development can be included in the school environment.
- In future studies, the effects of traditional games on character development can be examined or the effects of traditional games and educational games on character development can be compared.
- In addition to such studies, qualitative studies can be supported by taking student or parent opinions about game-based character education practices.

## Data Availability

The raw data supporting the conclusions of this article will be made available by the authors, without undue reservation.
